# Collectanea

**Published:** 1830-08

**Authors:** 


### COLLECTANEA.

Floriferis ut apes in saltibus omnia libant,
Omnia nos, itidem, depascimur aurea dicta.

### PHYSIOLOGY.

*A Female Pregnant who never Menstruated.* It has commonly been supposed that women who never menstruate are always barren: LINNÆUS is of this opinion. VELPEAU,* however, mentions an instance which occurred at the hospital of Tours, in which a woman, who had never menstruated, became the mother of a fine child. In general, the absence of the menses depends upon some malformation of the uterus, or the parts connected with it, and therefore is in most cases the sign of sterility.

* Art des Accouchemens. Paris, 1829.

*Case of Hernia of the Brain.* By Dr. Pierquin. (*Journal dès Progrès.*)

Rose ——, aged twenty-six years, of a lymphatic temperament, feeble constitution, and small stature, with blue eyes, light hair, was a servant in Romans, in the department of Drôme. When France was invaded by the enemy, the little town of Romans suffered greatly from the brutality of the soldiery. Some women were violated, among whom was the subject of this case. A short time after the attempt upon her person, Rose observed a white discharge, the existence of which she was afraid to communicate to any one. Her health grew worse daily, until at length she became a prey to constitutional syphilis. After having suffered the most excruciating, deep-seated and cephalalgic pains, she entered the hospital, at which time she had a venereal caries about the middle portion of the cranium. All the remedies usually employed in similar cases proved ineffectual. The caries increased daily, and the sanies discharged from it was so fetid that the patient suffered almost as much from it as from the disease. All the senses were unimpaired except that of smelling, and it could not be ascertained whether this resulted from the progress made by the disease, or from the odour of the discharge. It was in this desperate situation she was sent to the General Hospital at Montpellier, which she entered in 1821. The case, considered as the consequence of caries from a neglected venereal affection, offered in itself nothing highly interesting, but it was different when viewed in relation to its bearing upon the physiology of ideology. We at first endeavoured to ascertain the influence of the external air upon the circulation. During the evening we counted 120 pulsations, and from 98 to 100 during sleep. This number was varied by the agreeable or disagreeable nature of the dreams, as was ascertained upon the patient's waking: under the two last circumstances, the protrusion of the cerebral pulp became stronger, whilst it did not exist at all in undisturbed sleep. The first of these phenomena took place equally whilst awake, just as if, in the generation or conception of ideas, the brain was struck with a state of orgasm approaching a genuine erection. During quiet sleep the cerebral subsidence was such, that the organ seemed to repose upon itself, at which time the encephalic pulp withdrew entirely from the lips of the wound. Often at the dressing hour, the patient was not yet awake, and it became necessary to rouse her from ordinary long and sound sleep; the difficulty attending the passage from this cerebral inertia to activity, was marked by such a state of orgasm, that it became necessary at each dressing to take off a considerable portion of the pulp of the brain; an operation always unattended with pain, and unperceived by the patient, who seemed to suffer from it neither moral nor physical inconvenience. So many observations were doubtless sufficient to prove that the brain is the ideologic centre. But in addition to these, we very often subjected the patient to another conclusive experiment. Whilst placed on her seat during the dressing, we entered into a conversation upon some topic that might fix her attention. The moment she became engaged, the oscillatory movements of the brain became at once more rapid and stronger: pressure was now applied upon the brain as strongly as possible, and in an instant the patient lost the use of all her senses, could no longer form an idea, ceased to speak, terminating the conversation suddenly in the middle of a word, which she finished when we removed the compression. The same phenomena took place in regard to conversation commenced, the patient completing the phrase when we ceased to press upon the ideologic organ. These different experiments were not only unattended with the slightest pain, but were unknown to the patient, who never perceived the interruption to her intellectual existence, which we occasioned at pleasure.

From this curious case, we may conclude that the brain is the central organ of the sensations, and that without it there would be neither attention, perception, or memory.

### PATHOLOGY.

*Paralytic Cases, with Serous Effusion, or with slight Morbid Appearances.* (Dr. ABERCROMBIE *on the Brain.*)

When a paralytic attack of the most formidable appearance passes off speedily and entirely, without leaving any trace of disease, we may suppose that no very serious injury has been done to the substance of the brain; and that the disease bears a close analogy to the affection which I have termed Simple Apoplexy; or, in other words, that the cause had consisted of a state of the circulation of the brain, which is capable of being speedily and entirely removed. Many cases again are fatal, and present, on dissection, only serous effusion, often in small quantity. A man, mentioned by Morgagni, had palsy of the right arm, and died apoplectic in two days. On dissection, no morbid appearance was discovered, except serous effusion, both in the ventricles and on the surface of the brain. Another had loss of speech, and palsy of the left side, and died comatose at the end of a month. Considerable effusion was found on the surface of the brain, but very little in the ventricles. A third had loss of speech and palsy of the right side, then became comatose, and died in five days. The ventricles contained about two ounces of fluid; there was also a good deal on the surface of the brain, which appeared to be most abundant on the right side.

I have formerly given my reasons for believing that serous effusion in apoplectic cases is not a primary disease, but a termination of simple apoplexy; and I have described cases in which it existed to a considerable extent without paralysis. In the cases, again, in which it has been accompanied by paralysis, the quantity of fluid has borne no proportion to the symptoms, and has been equally distributed over the brain; except in the case now quoted from Morgagni, in which, too, it is worthy of remark, it appeared to be most abundant on the same side with the disease. From these considerations, I think we may conclude, that, in the cases now referred to, the effusion was not the cause of the paralysis, but the effect of the termination of a certain state of the circulation in a part of the brain, with which the paralysis had been connected from the first invasion of the disease. The whole phenomena of palsy do, indeed, bear evidence, that certain cases of it depend upon a cause which is of a temporary nature, and capable of being very speedily and entirely removed. We see hemiplegia take place in the highest degree, and yet very rapidly disappear; but the most singular circumstance connected with certain cases of palsy, is, that we occasionally see it continue without any improvement for many weeks or months; and then, from some change which entirely eludes our observation, take a turn for the better, and very suddenly disappear.

I do not know to what class I ought to refer the following case, but I shall introduce it here as the most remarkable example that has occurred to me, of long-continued palsy, without any satisfactory morbid appearance.

CASE I. A gentleman, aged 35, while standing in the street, conversing with a friend, suddenly lost his speech; he recovered it after a few minutes, walked home, and made no particular complaint. In the evening of the same day, he suddenly fell from his chair, speechless, and paralytic on the right side, but without coma; being sensible of what was said to him, and answering by signs. He was then confined to bed for several weeks without any change in the symptoms. At the end of three months, he had recovered so far the motion of his leg, as to be able to walk a little, dragging forward the leg by a motion of the whole right side of his body. He afterwards improved considerably in bodily strength, so that he could walk for several miles; but his thigh and leg continued to be dragged forward by the same kind of effort, without any further improvement. He never recovered any degree of motion of the arm or hand; he could not even move the fingers; his speech was very inarticulate and his countenance was expressive of great imbecility of mind. In this state he continued without relapse, and without any further improvement, for fifteen years, when he died at the age of 50. For a month before his death, he had been declining in strength. I saw him about four days before he died, and found him in a state resembling typhus; his pulse frequent and weak; his tongue very foul, and dry in the middle; he made no complaint. He was not then in bed, but was confined to it next day, and died in three days more, of rapid sinking without coma.

*Inspection.* The membranes adhered firmly to each other, and to the brain, at a spot the size of a shilling, on the upper part of the right hemisphere. There was a large quantity of fluid under the arachnoid membrane, and a considerable quantity in the ventricles. Near the posterior part of the longitudinal sinus, a small part of the sinus appeared to be thickened in its coats, and the inner surface of this part was dark-coloured and slightly fungous. The canda equina was of a remarkably dark colour, as if it had been soaked in venous blood, but without any change in its structure. No other morbid appearance could be detected, on the most careful examination, in any part of the brain or spinal cord.

The remarkably diseased state of the arteries of the brain, which has been already referred to, as a very common appearance in elderly people, may probably be the cause of temporary interruptions of the circulation, and slight paralytic attacks, which after some time may pass off from changes which elude our observation. Perhaps something of this kind occurred in the following case, for which I am indebted to Dr. Simson.

Case II. A gentlemen, aged 58, in 1805, was attacked with hemiplegia of the right side, without coma. After the usual treatment, he improved gradually; and at the end of five months he had quite recovered, except that his right leg continued rather weaker than the other. In 1812, he had another attack, preceded by violent headach. From this likewise he gradually recovered, though his recovery was much less perfect than after the former attack. He had four other attacks betwixt 1812 and 1817, which were carried off by the usual remedies, bloodletting and purging. In 1817, he had another and severe attack, likewise in the right side; his speech was unintelligible, and his mouth much drawn to the left side. He was relieved by bloodletting, &c. but from the effects of this attack he did not recover. He remained paralytic in the right side, his mouth twisted, and his speech very indistinct. In November 1818, he began to be affected with gangrene of the toes, which advanced slowly, and terminated fatally, in April 1819. From the time of his first attack, in 1805, to the second, in 1812, he always became confused and felt headach, when he attempted to read or write, so that he was obliged immediately to desist; but after the attack in 1812, he was able to read and write without any confusion or uneasiness, and even without the use of spectacles, which he had formerly employed.

*Inspection.* The dura mater seemed considerably thickened; the pia mater also seemed thickened, and was very vascular. There was extensive disease in the arteries of the brain, their coats being thickened, and in many places ossified. This was very remarkable in the principal trunks of the carotids and vertebrals, and was likewise traced into the smaller branches. The internal carotids seemed considerably larger than usual, and their coats were much thickened; and the vertebrals and their branches were affected in the same manner in a still greater degree, particularly about the tuber annulare, where the basilar artery was quite brittle, and gave off its branches in the same condition.

---

*Death from the Bite of a Viper.* By Dr. THOMAS, of Schlawe. *(Journ. der Pract. Heilkunde.)*

It has been erroneously asserted by FONTANA and other writers, that the bite of the European viper is never fatal to man. Many cases on record have already proved the contrary. Such accidents are particularly dangerous to children, because the virus of serpents acts in an inverse ratio to the size of the animal bitten. The following case occurred in Germany.

A girl, eleven years old, was bitten by a viper above the right internal ankle. Immediately afterwards, she felt a burning sensation throughout the limb, violent colic, and distressing thirst, followed by frequent vomiting. No remedy was administered; the patient only drank freely of water, to relieve her thirst. In three hours after the accident, symptoms of suffocation occurred, and the girl soon died. The body quickly passed into a state of putrefaction.

---

*Hydrops Pericardii.* SOEMMERING says that only a few drops of fluid are found in the pericardium in a natural state. TESTA has not seen it exceed a spoonful or two. CORVISART is not inclined to consider the secretion as morbid, *unless it exceed six ounces.* Sometimes, in dropsy of the pericardium, the fluid is collected in numerous small sacs, or hydatids, adhering either to the free portion of this membrane or to the heart.—*North American Med. and Surg. Journal.*

---

### SURGERY.

*Case of total Section of the Trachea and Œsophagus, cured by leaving a fistulous Opening for the continuance of Respiration.* By Dr. LUDERS. *(Revue Médicale.)*

A man, æt. 37, athletic and unusually strong, wishing to escape imprisonment for robbery, tried to cut his throat with a pruning knife. He was found lying on his belly, the head supported on his two hands; he had still strength enough to run some distance to the hospital, where he came some hours after the accident. The wound in the neck was three inches wide, and six long; it had evidently been made by repeated cuts; it divided transversely the trachea between the first and second rings, as well as the œsophagus, and reached the cervical vertebræ, without wounding either important blood-vessels or nerves; the upper muscles of the neck only were cut, the sterno-cleido partially. In order to inflict this wound, the man must have plunged the weapon deeply by the side of the trachea, and cut from within outwards. He was pale and speechless, and only able to breathe when lying on the belly, a position which permitted the blood to flow from the wound, in any other, breathlessness and threatening suffocation came on. Indeed, these symptoms having happened on attempting to examine the wound while sitting, he violently resisted all efforts again, and remained all night lying on his belly.

As soon as I was satisfied of the complete section of the œsophagus, considering the case hopeless, I pronounced that the man would die from inanition. I tried to pass into the lower end of the œsophagus an elastic gum canula, in order to throw in some warm milk; but violent vomitings and threatening of suffocation came on. As there was much thirst, the mouth was occasionally moistened with milk, which partly came out by the wound, and occasionally fell into the trachea, provoking excessive cough: it, however, calmed the feeling of thirst. He occasionally also sucked a slice of citron, and had an enema of gruel, with fifteen drops of Tinct. Opii.

Next day, I found a great change: the patient had not slept, and from time to time had thrown up, by a violent convulsive cough, quantities of coagulated blood mixed with mucus. The difficulty of breathing and stertor caused by the repletion of the bronchiæ were gone; the patient was lying on the back, and able to sit up. My plan, then, was to unite the two ends of the trachea by several stitches, and, after the union of the outer wound, to keep the head close to the chest by a proper bandage. A strong thread was passed through the lower fold of the wound, which was then raised close to the upper; but, on trying to join them, so strong a convulsive cough came on, that the danger of suffocation was imminent, the face becoming of a violet colour, and the patient throwing up his hands, stretching the neck till the lips of the wound were again sufficiently separated to allow of free breathing through the opening. I can only explain this by supposing the upper part of the trachea and larynx paralyzed, from the division of some of the twigs of nerves supplying them. After many other attempts, I was obliged to be satisfied with bringing the head close to the chest by means of a bandage, and carefully clearing the wound from mucus and blood.

The same day, quantities of bloody mucus continued to pass out of the wound; the pulse was calm and full; no fever. In the evening, he was hungry. I had a tin tube, in the form of S, with a large funnel at one end, and an ivory point at the other, so as to administer aliment by the wound. At present I was satisfied with an opiate enema, to blunt the feeling of hunger.

Third day.—Some sleep, without cough, and, excepting hunger, felt well; no fever. A cupful of milk and yolk of egg was thrown into the stomach, by means of the apparatus: this relieved hunger, but not the thirst, which was only allayed by milk taken into the mouth, and passing through the wound, (another proof of the opinion that thirst has its principal seat in the mouth and œsophagus.) Another attempt at union of the lips of the wound failed. In the evening, a lavement, as there was pain in the bowels, and no motion for three days.

Fourth day.—Slept better, and felt well; breathing freer; and he observed that, in taking milk into the mouth, a portion of it passed into the stomach when he pressed violently the head against the chest. The wound was covered with a thin coating of lymph, without the appearance of inflammation or suppuration. Another attempt at union again brought on threatening of suffocation.

Fifth day.—The power of swallowing improves; the outer wound diminished in size; the patient swallowed a cupful of barley water and milk, the greater part of which reached the stomach. The introduction of the food by means of the tube always excited violent vomiting and cough. Small, slow pulse. A laxative lavement had no effect.

Sixth day.—Bad night; great agitation; violent hoarse cough, which only rarely expelled some puriform mucus through the outer wound; breathing short, hissing, and accelerated; anxious look of the countenance. The wound looks well, and is covered with lymph. Notwithstanding these appearances of bronchitis, the exhaustion of the patient from loss of blood and want of nourishment; the state of the pulse, which varied much, sometimes full and hard, then small and soft, but generally frequent; I suspected rather a spasmodic state than inflammation. The patient disliking milk, warm water was substituted; a lavement given; a sinapism on the chest, and a warm bath.

Seventh day.—All symptoms calmed; the patient bathed in perspiration, smelling of sour milk; urine clear, like whey, and smelling like perspiration, (yet giving no indication of acidity.) These two qualities of the sweat and urine continued while the patient lived only on milk. The discharge from the bronchiæ continues, and there is much difficulty in removing the mucus which accompanies the bleeding of the wound. The power of swallowing by the mouth increases.

Eleventh day.—Cough diminished. For the first time, the patient today ate some soup with bread, of which very little came through the wound, causing it to bleed and provoking cough. The wound of the œsophagus was not to be perceived, and thus nature alone had done what art could not. The lower lip of the trachea is a good deal raised, so that the distance here is about an inch from the upper, and parallel to the lower margin of the wound of the integuments. At this stage the little irritability of the respiratory organs might have permitted a junction by suture, in the case of there being no paralysis of the upper part, if that operation had not been interdicted by the change that had taken place in the œsophagus, the substance of which would have been torn by the stretching necessary; and thus the most difficult part of the cure again undone. The only question, then, was to direct the cicatrization so that a substance of new formation should fill the gap of the trachea. The chief difficulty appeared to be to hinder the granulations from springing up in excess, and closing the windpipe. To prevent this, I determined to continue the same diet, to give only liquid food, simple and in small quantity, and chiefly milk: in fact, during fifteen days, milk, occasionally mixed with yolk of egg, was his only food. Under this regimen, the pulse came down to forty, the cough lessened, the wound gradually healed; there was a motion every fifth or sixth day, by means of lavements. The patient was able to sit alone, but not to walk.

Seventeenth day, (March 6th.)—The posterior and lateral sides of the trachea were perfectly formed, yet, when the outer opening was closed, the patient could neither breathe nor speak, and very shortly a feeling of suffocation came on. This was not caused by the shutting up of the larynx or glottis; for a sound, introduced into the wound, came out through them: we must suppose it to arise either from paralysis of the larynx, by the lesion of some of its nerves, or from want of exercising the part. However, I determined to persevere in uniting the external wound, (which was now a round opening, about eight lines in diameter,) as long as there was no difficulty of breathing, and the inner part of the trachea kept free from fleshy granulations.

March 12th.—The patient begins occasionally to breathe through the nose; but suddenly a suffocating cough came on, with hissing breathing, and expectoration of mucus mixed with blood. These symptoms were occasioned by the filling up of the lower part of the trachea by granulations, so that the air could hardly enter from the outer wound. The introduction of a sound removed them, and the granulations were kept down by caustic.

15th.—The breathing has been free till now, when similar symptoms to those on the 12th recurred, and were removed by the same means. It appeared to me necessary, both for the purpose of repressing the granulations and keeping open the passage for the air, to introduce a leaden canula accurately fitted.

March 17th, this was done without trouble, and was worn several weeks without causing any essential change; only the patient, by degrees, took food more solid and nourishing. The external wound formed a round opening moulded on the canula, discharging a little serum, from the irritation caused by the daily removal and replacement of the canula.

In fifteen days after the introduction of this instrument, the opening of the upper part of the trachea and larynx continued pervious, and the patient occasionally breathed through the nose when the canula was out. This was withdrawn, for the purpose of trying again to close the external wound, but at the end of four days, the canal being almost closed by the granulations, it became necessary again to introduce it, to prevent suffocation: however, a round hole was made in the canula opposite to the lower orifice of the larynx, so as to habituate him to breathing through the nose, and possibly to reestablish the power of speech.

In fact, on the 5th of April, when the patient closed the canula with the finger, he could not only breathe by the nose, but even speak, though in a thick and dull voice. I conceived hopes of a complete cure; but all attempts at establishing natural breathing without the canula failed. A cork was put in the canula, but the patient could only keep it there a few hours, so much mucus accumulated, which he could not free himself from by spitting up, and the dyspnœa became intolerable. This mucous secretion rendered abortive all means tried to do without the canula: the only thing left was to render the use of this tube less irksome. A silver one was substituted in its place, which constantly remained, while a second one, made to fit it, could be removed and cleaned as often as required. A thin gauze was worn over the opening, to prevent extraneous substances from passing in. It is now two years, and the man is well. Sometimes he has cough, and bleeds a little at the opening in the neck, caused possibly by the irritation of dust inhaled.

*Reflections on the above case.* PARÉ saw a man who, with the stroke of a poignard, had completely divided the trachea and œsophagus, so that the latter had shrunk so low that it could not be laid hold of. Although the wound of the trachea was united by suture, the patient died four days after the accident; but he had previously recovered his voice, and could tell his murderer. Paré supported him by giving analeptic lavements, and holding under his nose fresh bread steeped in wine.* This case proves the possibility of a complete section of the two canals, without the wounding of large vessels or nerves, and shows that the accident may not prove immediately fatal. PLACENTIUS† speaks of the simultaneous lesion of the trachea and œsophagus, in an attempt at suicide, without wounding the jugular veins: the man was cured in a month. S. HELEVIZ‡ has a similar case, where the man survived ten days. PURMANN§ describes a wound made by a sharp instrument, by which the jugular vein and the trachea and œsophagus were wounded, and the throat cut obliquely across. After vainly trying to stop the hemorrhagy, he tied the vein. The wounds in the two canals were healed in four weeks, that of the neck in twelve. STARCK, DEBRUCK, HURTSNIG, MURSINNA, and RUST, describe cases of a similar nature where cures were effected, the treatment being very simple, bringing the head close to the chest, supporting life by lavements, &c.

It results from the case, the particulars of which have been described,

1st. That a complete section of the trachea and œsophagus, without injury to the great blood-vessels or nerves, may be caused by an attempt at suicide; but it cannot take place unless the cut be made by means of a curved knife, plunged in by the side of the trachea, and then drawn from within outwards. Death by cutting the throat is one of the most difficult problems of legal medicine, when one must decide, from the appearance of the dead body, whether suicide or murder has taken place. The above case proves that the complete section of the two canals is not necessarily mortal. I have no doubt but, under more favorable circumstances, it might even be cured without leaving any permanent inconvenience.

2d. This case shows the healing powers of nature: here nature not only preserves life, but reestablishes so far the œsophagus, that it is able perfectly to resume its functions. The cure was entirely her work. However, the diet, at first from necessity, then designedly prescribed, and the use of warm milk only, had contributed a good deal towards this rare result, that a large wound of such irritable organs, continually exposed to the air, and in a vigorous and sanguine subject, should be cured without an inflammation requiring the application even of a single leech, and without a single drop of pus being formed in the progress of cicatrization. For the first ten days, the wound was kept constantly moist with the warm milk which was swallowed. It appears to me that milk, as an animal liquor, is the best fomentation for an abraded surface. With poultices of milk, and low diet, the worst-looking ulcers are healed sooner than with excitants, &c. In general, low diet has not the credit which it deserves in the treatment of chronic diseases, external as well as internal. The state of the sweat and urine in this case proves to what extent it acts in altering the organizations of the body.

The ease with which, in the case described, the trachea suffered the presence of a foreign body, and the quickness with which it accustomed itself to it, show that one may frequently have recourse to this as a means of relief. This fact would call the attention to tracheotomy in cases of croup, when it has its seat exclusively in the larynx and trachea.|| The great point is to gain

* Paré, l. iii. c. 29.

† Chir. l. xx. Obs. Med. rar. a S. Schenck, Francfort, 1400, l. ii. p. 247.

‡ Obs. Phys. Med. Aug. Vind, 1680. Obs. 63, p. 210.

§ Cur. Chir. Obs. 1710. Obs. 16, p. 73.

|| But can we ever determine, during life, in what part croup commenced, or to what parts the inflammation is limited? Mr. PORTER, whose work on time. Here the child does not die by the mechanical shutting up of the trachea, but of the glottis: generally, the cause of death is the exhaustion of the vital principle; congestions of black and liquid blood in the lungs, the right side of the heart, and the sinuses of the brain, and, what lengthens the agony of dying, an abundant exudation into the pericardium and ventricles of the brain. These phenomena are more frequently found on dissection, than the existence of exudation in the air-passages, and occur from the first moment of the inflammation by the spasmodic closure of the glottis. I think I am able, by numerous facts, to prove that the membranous exudation is less frequent than generally supposed. As soon, then, as the symptoms of want of oxydation of the blood occur, and which are generally considered those denoting exudation, but which indicate only the approach of death, as the earthy colour of the face, the blueness of the lips, the difficulty of breathing, the anxious look, the disposition to throw back the head, and the pulse small and frequent; then is the time to perform tracheotomy, and to leave a silver canula in the trachea, not only to prevent the fatal result, but also to give time to try the means proper for subduing inflammation, or to procure the exit of the false membrane.

It appears to be a subject worthy of consideration, whether, in cases of laryngeal phthisis, the placing a canula in the trachea would not be advisable. M. Luders has generally found, on opening subjects dying of this disease, a state of chronic inflammation of the larynx, and a secretion of puriform mucus, with swelling and thickening of the mucous membrane; and death has been caused by suffocation from closure of the glottis.

---

*Aneurism of the Arteria Innominata, involving the Subclavian and the Root of the Carotid, successfully treated by tying the Carotid Artery.* By Valentine Mott, M.D. Professor of Surgery, New York.

Moses R. Gardner, ætat. 51, by profession a farmer, of sound constitution and good habits of life, applied to me some time in March for advice.

He gave the following relation of his case: About three years ago, while occupied in removing a building, and compelled to lift heavy weights, he was attacked with pain in the upper and back part of the neck. This lasted until the month of January, when it extended to the right shoulder and arm, and continued until the following May; it then partially subsided, and he observed his voice was becoming hoarse, which he attributed to exposure and consequent cold. About eighteen months since, while shaving, he discovered a small swelling at the upper part of the breast bone, but did not remark any the surgical pathology of the larynx and trachea deserves attention, well observes, in reference to the question of performing the operation of bronchotomy in cases of croup, that "the effusion of coagulated lymph is very generally confined to the larynx alone; but still, in a number of cases, the inflammation commences in the bronchial cells, and proceeds upwards in the windpipe. This is an affection in which an operation could not possibly be of service, and there is no mode of distinguishing accurately as to what has been the original seat of the disease. This one consideration must involve every case in obscurity, and render the success of an operation a matter more dependent on chance than on judgment." (P. 48.) An analysis of Mr. Porter's excellent work is contained in our Journal for November 1827, p. 439.—Editor.

throbbing in it until some time afterwards. He had consulted a physician, but received no positive opinion on the case.

Upon examination, I found above the sternum a pulsating tumor, about the size of a pigeon's egg, spreading some distance under the clavicular and sternal portions of the right sterno-mastoideus muscle, in the course of the subclavian artery, and extending as low down upon the pleura as the second rib, compressing more or less the bronchial tubes, and producing, on the least coughing or exercise, a wheezing, not unlike that of asthma. He shrunk from the least pressure upon it; complaining of impeded respiration, followed by pain. Its pulsations were synchronous with those of the heart, and decidedly aneurismal.

After fully explaining to him the nature of his disease, and its probable fatal termination should it increase and be left to itself, I advised him to return home; to avoid all exertion; to be occasionally bled, and to confine himself principally to a vegetable diet; but should he observe the least increase either of the tumor or any of his symptoms, to come again to me, and I would decide on the propriety of an operation.

After that time I occasionally saw him; he seemed to understand his case fully, and was very desirous to take the chance of the operation: but, as I could not observe any material change in the disease, I recommended him to pursue the same directions, and wait patiently until it should occur.

On the 12th of September, he again came to the city. I found the tumor above the sternum had increased to the size of a large walnut, and upon a careful application of the stethoscope, it was evidently encroaching more upon the chest. The whizzing sound (*bruit de soufflet*,) could be heard; the thoracic viscera were sound, the respiratory murmur being distinct throughout. His respiration was very much impeded by speaking, walking, or coughing, and almost entirely suspended by the least pressure upon the tumor; the action of the right carotid was much more feeble than that of the left; no pulsation could be discovered in its branches; the right subclavian, external to the scaleni muscles, was natural, while the axillary and brachial arteries could hardly be felt; at the wrist no pulse could be found; the pulsations of the arteries of the left side were natural. His general health was good.

In reflecting upon this case, and comparing the relative situation of the parts, I was pursuaded the aneurism was of the arteria innominata, involving the subclavian and the root of the carotid.

I thought further delay unnecessary, and he being willing to abide by my judgment, after having stated to him the chances of the operation, I resolved on its performance. From the evident interruption in the circulation of the right arm, and the apparent effort of nature to effect a spontaneous cure, I determined upon tying the carotid first, to observe the result, and afterwards to secure the subclavian, should it be required.

On the 26th of September I operated. The artery was taken up in the usual manner; no material change was observed.

27th, nine A.M.—Slept well, and feels refreshed; thinks there is more room, as he expresses it, in breathing; complains of a little soreness of the tonsils in swallowing; pulse fifty-eight, regular, and tranquil; skin natural, pulsation and size of the tumor evidently diminished.

Nine P.M—Much more restless from mental alarm; pulse fifty-eight, tense. In other respects, the same as in the morning; being habituated to laudanum, was permitted to take a teaspoonful.

28th, nine A.M.—Slept well after the opiate; breathes easily, and says he takes "a more satisfactory breath" than he did before the operation; feels much less of the pulsation in the tumor; pulse sixty-three, not so tense; skin natural; cough much less. Ordered a dose of calcined magnesia and Epsom salts.

Nine P.M.—Has passed a comfortable day; his wife, who arrived from the country since the morning, expressed her surprise at the improvement in his voice and breathing; and the difference in the beating of the tumor. Pulse of the right radial artery very distinct, but intermitting once from ten to fifteen beats; in the left arm, eighty; coughs frequently, and expectorates freely; skin natural; tongue a little white; salts have not operated. Ordered the dose to be repeated, and, if restless after its operation, to take his usual anodyne.

29th.—Saluted me this morning upon entering his room, with a full and fine voice, and said he was well enough to call on me; salts operated freely; thinks his cough and expectoration much less. I found him lying down, and breathing quietly; pulse seventy-one, and regular. The radial artery of the right arm beating as last evening, with fewer intermissions, but of longer continuance; skin over the tumor more wrinkled; pulsation appears less, and feels weaker. Directed to continue his tea, toast, and gruel.

Eight o'clock.—As well as in the morning; takes a full breath without the least wheezing; pulsation in the wrist very distinct and regular; in the left sixty-two to the minute. Continues the opiate.

30th.—Found him lying more recumbent than at any former period; pulse seventy, and regular; right radial artery does not beat quite so firm as yesterday; wound discharging a little, was dressed.

October 2d.—Says he now feels as if he would get well; cough rather more troublesome; pulse fifty-seven; pulsation of the right radial the same; his bowels not being free, directed Submur. Hydr. grs. viij.; Sup. tart. Potassæ, Pulv. Jalapæ, āā ℈j. M. Evening. Medicine has not operated; directed a dose of Sulphate of Magnesia.

3d.—Cough and bronchial effusion very much diminished by the operation of the cathartic; pulse sixty-eight.

4th.—Feels very well; passed a good night; all his symptoms improved; pulse seventy-four; can bear any degree of pressure upon the tumor without the least pain or difficulty of breathing.

10th.—Continues to mend, and is sanguine as to his recovery; pulsation of the tumor hardly perceptible, and to the touch very much diminished; cough less troublesome; left pulse sixty-six; right, very feeble.

16th.—Ligature separated and came away last night; the tumor above the sternum, and pulsation, entirely disappeared; cough and breathing better; voice nearly natural; pulse sixty-six; now and then a very faint pulsation of the right radial artery; right hand a little swelled, and feels numb, and complains of the want of power to close it.

22d.—Wound just healed; weakness of the arm very considerable; fingers very thick and clumsy; arm swelled, and pits upon pressure; no pulse in the right radial artery; breathing very easy; cough and expectoration much less; can sleep easy in any position, which he has not been able to do for many months.

26th.—Left town this morning for his residence in New Jersey. (Condensed from the *American Journal of Med. Sciences.*)

